# Aerobic exercises and cognitive function in post-stroke patients: A systematic review with meta-analysis

**DOI:** 10.1097/MD.0000000000031121

**Published:** 2022-10-14

**Authors:** Xiaogang Li, Di Geng, Siyue Wang, Guotao Sun

**Affiliations:** a Chengdu Sport University, Chengdu, Sichuan Province, China; b Sichuan University of Science & Engineering, Zigong, Sichuan Province, China; c Sichuan Tourism University, Chengdu, Sichuan Province, China; d Yangtze University, Jingzhou, Hubei Province, China.

**Keywords:** aerobic exercise, cognitive function, meta-analysis, post-stroke patients

## Abstract

To study the effect of aerobic exercise on cognitive function of post-stroke patients, especially to identify specific interventions that the most likely to maximize cognitive benefits of stroke patients.

**Methods:**

According to the PRISMA principle, the databases of Web of Science, EMBASE, PubMed and Cochrane library were searched to collect randomized controlled trial data of aerobic exercise on cognitive function intervention of post-stroke patients. The Cochrane bias risk evaluation instrument was used to assess the methodological quality of included studies. Review Manager 5.4.1 software was used to analyze heterogeneity and potential publication bias.

**Results:**

A total of 11 criteria studies that satisfied the association between aerobic exercise and cognitive function following stroke were selected to be included in the review. Global cognition ability was significantly improved after aerobic exercise intervention (0.51; 95% confidence interval [CI] 0.16–0.86; *P* = .004), moderate intensity had the largest effect size on improving global cognition ability (0.98; 95% CI 0.48–1.47; *P* = .0001), none of cognitive flexibility, working memory, selective attention and conflict resolution showed the significant difference from zero.

**Conclusion:**

Aerobic exercise has a good impact on enhancing the cognitive dysfunction of patients after stroke, which stroke patients were found to benefit the most from moderate-intensity exercise. However, our studies did not found that aerobic exercise had an active result on cognitive flexibility, working memory, selective attention and contention resolution.

## 1. Introduction

The stroke is an acute cerebrovascular disease with high morbidity, disability and mortality, and its common symptoms are declining in motor skills and sensory function, vision loss, aphasia and cognitive impairment.^[1]^ Stroke is one of the main factors of death and disability in the global population and a serious threat to the public health development of the global population.^[2]^ With the increasing aging of the population, the incidence rate of stroke has shown an increasing trend. Stroke not only brings personal pain to patients, but also brings greater economic burden to patients’ families and society.^[3]^ Stroke survivors suffer from stroke illness 1 year with their cognitive impairment at a rate of as high as 38%, mainly with decreased brain functions such as understanding, memory and attention. Stroke critically harms patients’ cerebrovascular health, and may deteriorate into vascular dementia if not treated in time.^[4,5]^ Although drugs can improve the cognitive impairment of stroke, long-term medication will produce many side effects and expand the economic burden of patients.^[6]^ Therefore, it is necessary to analyze effective alternative therapy strategies. In recent years, many scholars have conducted multidisciplinary research on cognitive impairment after stroke, among which exercise intervention research is also increasing. Due to the different experimental design, sample size, and measurement tools, these research results are also different. It is demonstrated by some experiments that exercise intervention can enhance the cognitive impairment of stroke patients. As proved by Moore et al,^[7]^ exercise therapy improves cognitive function following stroke, Zheng et al^[8]^ found that regular Baduanjin training was associated with less cognitive loss in patients after stroke, Marzolini et al^[9]^ demonstrated that the combination of aerobic exercise and resistance exercise led to the improvement and reduction, of cognitive function in proportion to the threshold standard meeting of patients with mild cognitive impairment (MCI). In addition, there are some meta-analyses that indicate that physical training can positively affect post-stroke cognition with small-to-moderate treatment effects that were obvious even in the chronic stroke stage.^[10,11]^ Contrary to the above research conclusions, Ada Tang et al^[12]^ carried out high-intensity and low-intensity exercise intervention for 6 months in patients with cognitive impairment after stroke, and the results showed that their cognitive function and special executive function were not effectively enhanced, Gjellesvik et al^[13]^ found no benefit of high-intensity interval training on cognitive function of patients after suffering from the stroke. There are various types of physical activity, such as aerobic, resistance, and stretching exercise. Because each exercise has its own set of characteristics, it may have different effects on brain structure and function.^[14]^ Therefore, more research is required to indicate the effects of different types of physical activity programs on cognition in stroke survivors. A previous systematic review have demonstrated that the evidence that physical training improves cognitive function in stroke sufferers is comparatively restricted, and no research has pointed to the optimal intensity or frequency of aerobic exercise in stroke survivors.^[15]^ Since, the publication of the study in 2016, the number of relevant trials investigating the intervention of aerobic exercise on cognitive impairment after stroke has increased significantly.^[16-18]^

Therefore, it is essential to integrate current research results and experimental data in order to quantitatively integrate relevant experimental conclusions. Here, we will systematically review the impacts of aerobic exercise on cognitive function following stroke, in particular to identifying specific interventions to maximize cognitive gains, and providing evidence-based medical evidence for public health policy makers and exercise recommendations.

## 2. Method

### 2.1. Literature search

This study searched the data of the electronic databases Web of Science, EMBASE, PubMed and Cochrane Library from their establishment to June 8, 2022. The retrieval method is as follows: stroke (e.g., “Cognitive Dysfunctional” or “Cognitive Function” or “Cognitive Impairment” or “Cognitive Decline” or “Mental Deterioration” or “Memory” or “Executive Function” or “Language Disorder” or “Attention” or “Agnosia”); exercise (e.g., “Endurance Training” or “Physical Activity” or “Physical conditioning”); combinations of MeSH Terms and Title/Abstract will be used.

### 2.2. Study selection

The inclusion criteria were: A randomized-controlled experiment of cognitive impairment following a stroke. In comparison with the control group, the experimental group only increased aerobic exercise intervention regardless of the exercise style or intensity. In order to avoid deviation of experimental results due to too little time of exercise intervention, continuous aerobic exercise is required to be no less than 8 weeks, and exercise more than once a week. Included a validated neuropsychological test of cognition, experimental results on overall cognitive ability and its specific cognitive domain (e.g., memory, processing speed, etc). The experimental data are complete, the sample size, mean and standard deviation are provided, or standard error and 95% confidence interval (CI) of mean within the group. The exclusion criteria were: Duplicate literature. Including qualitative research, review and meeting summary. Subjects excluded patients with Parkinson’s disease, Alzheimer’s disease, dementia and other neurological disorders, as well as other pathology or disorders that affect exercise. Non-english literature.

### 2.3. Data extraction

According to the needs of the study, 2 searchers (XGL and GTS) extracted and entered various data of the included literature in an independent and double-blind manner, including: the first author of the literature, the year of publication, the sample size of the experimental group and the control group, gender, the age of the subjects, the intervention scheme (content, intensity, time, frequency and cycle), and the outcome indicators. If the studies included in the systematic review did not mention the intensity of aerobic exercise, we will refer to the classification criteria in Garber et al study^[19]^ to determine the intensity.

### 2.4. Quality assessment

The methodological quality of incorporated researches will be evaluated adopting the Cochrane Bias Risk Assessment Tool by 2 participants of the research group (XGL, DG). There are 6 types of bias, including selection bias, performance bias, detection bias, attrition bias, reporting bias and other bias. Risk of bias and quality of evidence were independently assessed by 2 members of the research team (XGL, DG). According to the risk level, 5 or more items are identified as low risk of bias; 3 to 4 are identified as moderate risk of bias; 3 or less are identified as high risk of bias.

### 2.5. Statistical analysis

We will apply the Review Manager software (V.5.4.1) from the Cochrane collaboration for data analysis. Continuous outcomes were summarized using standardized mean difference with their 95% CIs. Heterogeneity will be assessed using the *I*² statistic. For studies that had good homogeneity outcomes (*I*² < 50%) we will utilize fixed effect models. If 50% ≤ *I*² < 75% and the CI overlaps with the visual inspection of the forest map, we will use a random-effects model. If *I*² ≥ 75% indicates that there is too much heterogeneity between studies, we will only use a general description of the statistical results. When *P* < .05, there was a statistically significant difference between the 2 groups, demonstrating that the meta-analysis was statistically significant. If the number of included studies is more than 10, egger test and funnel plots were used to detect publication bias.

### 2.6. Ethical review and informed consent of patients

This research is a systematic review, so the content does not involve ethical review and unethical projects.

## 3. Results

### 3.1. Study selection

**Figure 1.**
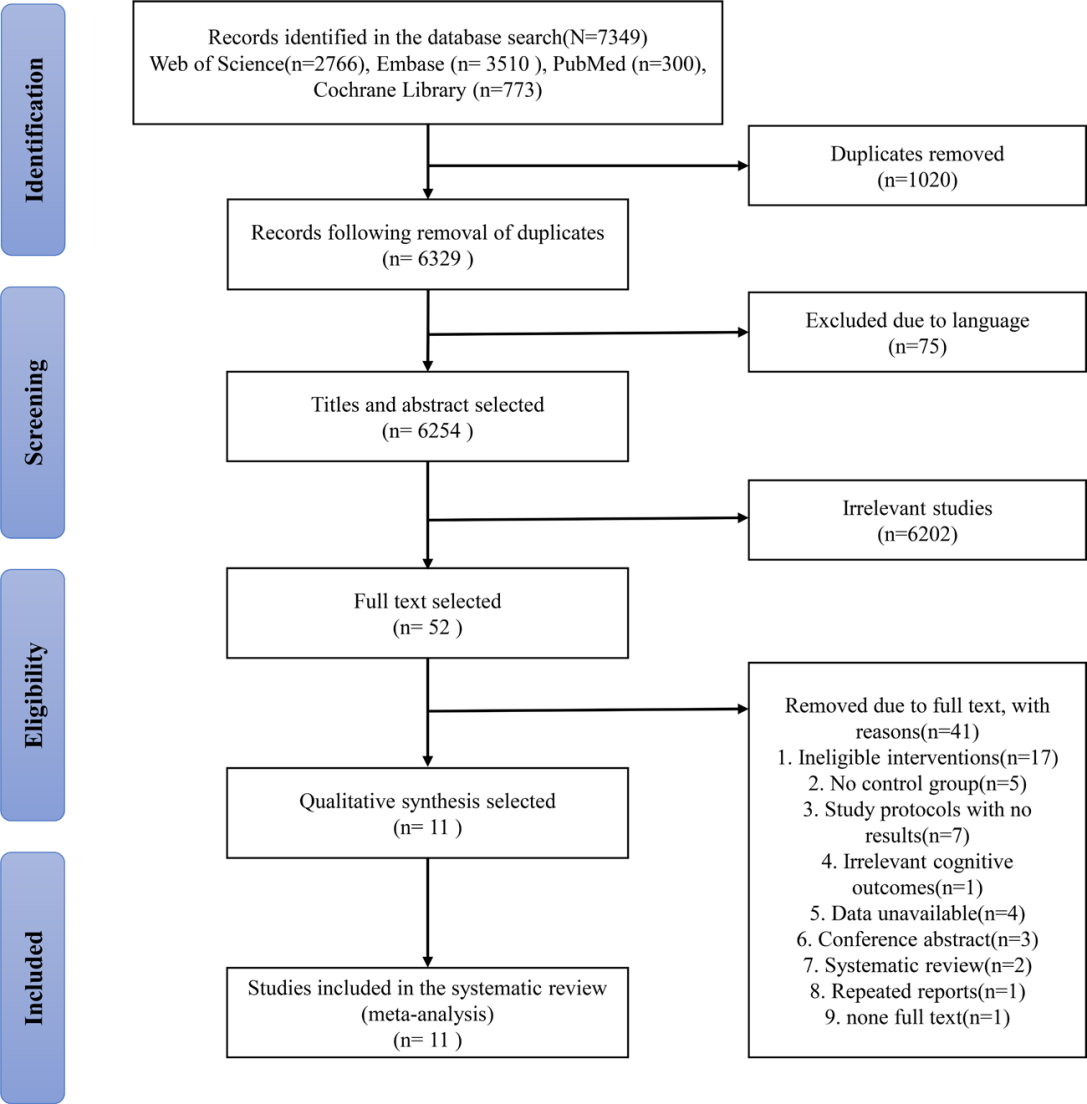
Flow chart of study inclusion and exclusion process.

In the primary search, 2766 results were retrieved from the Web of Science, 3510 from Embase, 300 from PubMed, and 773 from Cochrane Library. All records will be imported into EndNote V. X9 for screening, 1020 records were removed because they were duplicates. After preliminary screening by reading the headline and abstract of the documents, 6277 irrelevant literature were excluded, and there are still 52 documents left. After further reading the whole passage, 41 literature were excluded. Finally, 11 articles were included in meta-analysis (Fig. 1).

### 3.2. Study characteristics and quality assessment

**Figure 2.**
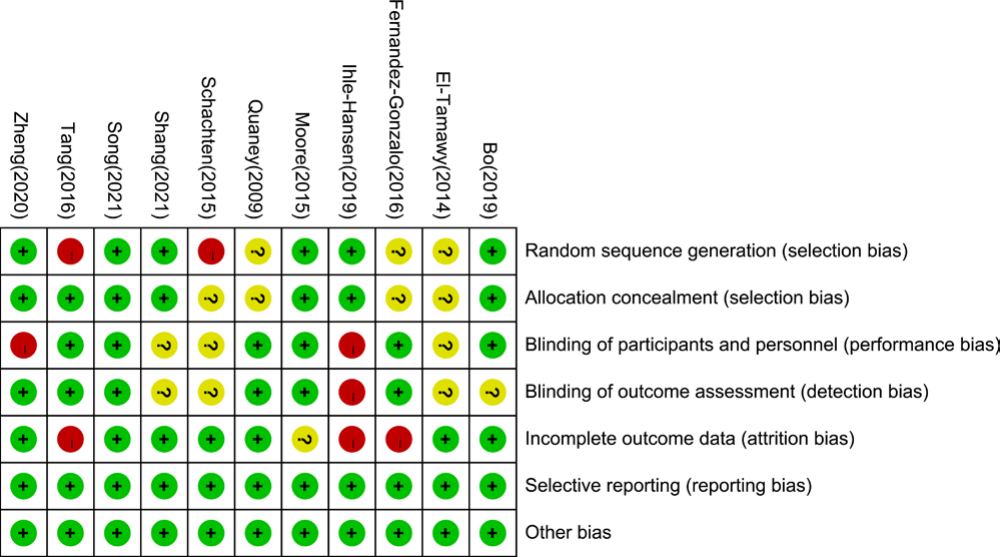
Risk of bias summary.

A detailed description of key characteristics for the 11 studies^[7,8,12,20-27]^ incorporated is given in Table 1. There were 824 subjects, 405 in the experimental group and 419 in the control group. Most of the subjects in both groups were male. In each study, subjects were randomly assigned to the experimental or control group. The intervention time range of each research was primarily 40 to 60 minutes, and the intervention frequency was 2 to 3 times/week, but the intervention period was very varied, with the shortest period being 8 weeks and some as long as 72 weeks. In terms of cognitive function measurement, included literature mainly adopts objective measurement or scale measurement, and the main indicators include overall cognition or specific cognitive areas, such as working memory, processing speed, selective attention and conflict resolution. The Cochrane bias risk assessment tool was used to assess the quality of the above literature, and the evaluation results were shown in Figure 2.

**Table 1:** Characteristics of included studies.

Study	Sample size (male/female)	Age (yrs)	Intervention	Time, frequency, duration	Intensity	Outcome/measurement
Song et al (2021)	e.g.=18(10/8)	e.g.=58.72 ± 17.13	Tai Chi	50 min/session, 2 session/wk, 6 mo	Low-intensity	Global cognition/MoCA, MMSE
	CG = 16(11/5)	CG = 57.18 ± 10.65				
Shang et al (2021)	e.g.=37(21/16)	e.g.=63.68 ± 9.42	Intensive grip training	50 min/session, least 3 session/wk, 12 wks	Unspecified	Global cognition/MoCA
	CG = 39(20/19)	CG = 64.13 ± 9.48				
Zheng et al (2020)	e.g.=24(19/5)	e.g.=61.63 ± 9.12	Baduanjin	40 min/session, 3 session/wk, 24 wks	Moderate-intensity	Global cognition/MoCA; Cognitive flexibility/TMT B; Memory/AVLT; Attention/TAP; Visuospatial ability/CDT;
	CG = 24(22/2)	CG = 62.75 ± 6.41				Processing speed/DSC
						Activities of daily living/MBI
Ihle-Hansen et al (2019)	e.g. =177(99/78)	e.g.=71.4 ± 11.3	Physical exercise	30 min daily physical activity and 45-60 min physical exercise including 2-3 bouts of vigorous activity every week, 18 mo	High-intensity	Global cognition/MMSE;Processing speed/TMT A;
	CG = 185(120/65)	CG = 72.0 ± 11.3				Cognitive flexibility/TMT B
Bo et al (2019)	e.g. =42(23/19)	e.g.=65.12 ± 2.56	Endurance, strength, and balance	50 min/session, 3 session/wk, 12 wks	Moderate-intensity	Cognitive flexibility/TMT B;
	CG = 47(27/20)	CG = 64.36 ± 2.31				Selective attention and conflict
						resolution/Stroop test;
						Working memory/DST Forward;
						Spatial imagination/MRT
Tang et al (2016)	e.g. =25(14/11)	e.g.=66 ± 7	Individualized exercise program	60 min/session, 3 session/wk, 6 mo	High-intensity	Working memory/DST Forward;
	CG = 25(15/10)	CG = 64 ± 10				Cognitive flexibility/TMT B;
						Selective attention and conflict
						resolution/Stroop test;
**Study**	**Sample size (male/female)**	**Age (yrs)**	**Intervention**	**Time, frequency, duration**	**Intensity**	**Outcome/measurement**
Fernandez-Gonzalo et al (2016)	e.g.=14(11/3)	e.g.=61.2 ± 9.8	Flywheel resistance training	4 sets of 7 maximal repetitions, 2 d/wk, 12 wks	High-intensity	Processing speed/TMT A;
	CG = 15(11/4)	CG = 65.7 ± 12.7				Cognitive flexibility/TMT B;
						Working memory/DST Forward;
						Selective attention and conflict
						resolution/Stroop test;
Schachten et al (2015)	e.g. =14	e.g.=55.14 ± 17.64	Golf training	60 min/session, 2 session/wk, 10 wks	Unspecified	Working memory/BBT;
	CG = 14	CG = 53.14 ± 13.54				Mental rotation ability/MRT;
Moore et al (2015)	e.g.=20(18/2)	e.g.=68 ± 8	Stretching and balance training	45-60 min/session, 3 session/wk, 19 wks	Low-intensity	Global cognition/ACE-R
	CG = 20(16/4)	CG = 70 ± 11				Activities of daily living/SIS
El-Tamawy et al (2014)	e.g.=15(11/4)	e.g.=48.4 ± 6.39	Bicycle training	40-45 min/session, 3 session/wk, 8 wks	Moderate-intensity	Global cognition/ACE-R
	CG = 15(10/5)	CG = 49.67 ± 6.98				
Quaney et al (2009)	e.g.=19(10/9)	e.g.=64.10 ± 12.30	Bicycle training	45 min/session, 3 session/wk, 8 wks	Moderate-intensity	Selective attention and conflict
	CG = 19(7/12)	CG = 58.96 ± 14.68				resolution/Stroop test;
						Cognitive flexibility/TMT B-A

ACE-R = Addenbrooke’s cognitive examination revised, AVLT = auditory verbal learning test, BBT = block-tapping task, CDT = test of attention performance, CG = control group, DSC = digit symbol coding, DST = digit span test, EG = experimental group, MBI = modified Barthel index, MMSE = mini-mental state examination, MoCA = Montreal cognitive assessment, MRT = mental rotation test, SIS = stroke impact scale, TAP = test of attention performance.

### 3.3. Data synthesis and meta-analysis

#### 3.3.1. Global cognition ability.

**Figure 3.**
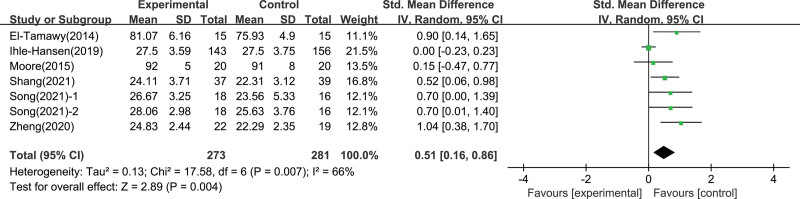
Forest plot showing individual study and pooled effect of aerobic exercise on global cognitive function.

A total of 6 literature studies evaluated the intervention effect of aerobic exercise on global cognition ability. Song et al‘s research used 2 measurement tools, Montreal Cognitive Assessment (MoCA) and Mini-Mental State Examination (MMSE). Homogeneity test was performed on the involved literature (*I*^2^ = 66%, *P* = .007), indicating moderate heterogeneity among multiple studies, so the random effect model was adopted. Global cognition ability was significantly improved after aerobic exercise intervention (0.51; 95% CI 0.16–0.86; *P* = .004), which was statistically significant compared with the control group (Fig. 3).

**Figure 4.**
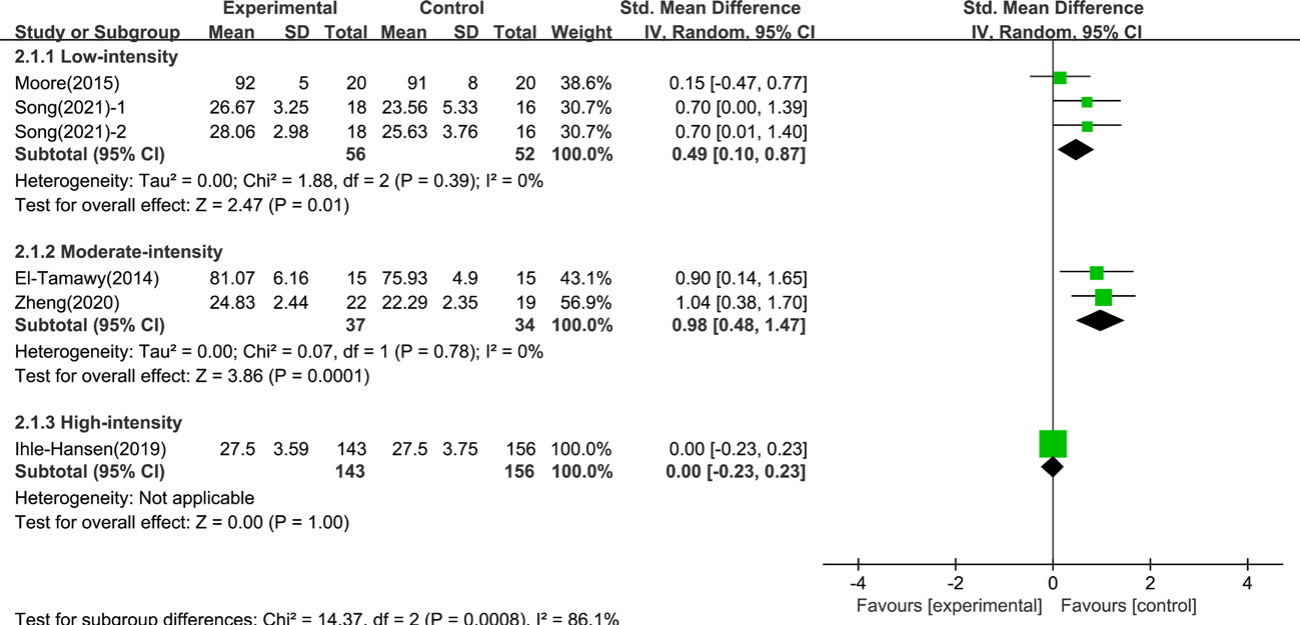
Subgroup analysis of global cognitive function effect size under different intensity of exercise.

**Figure 5.**
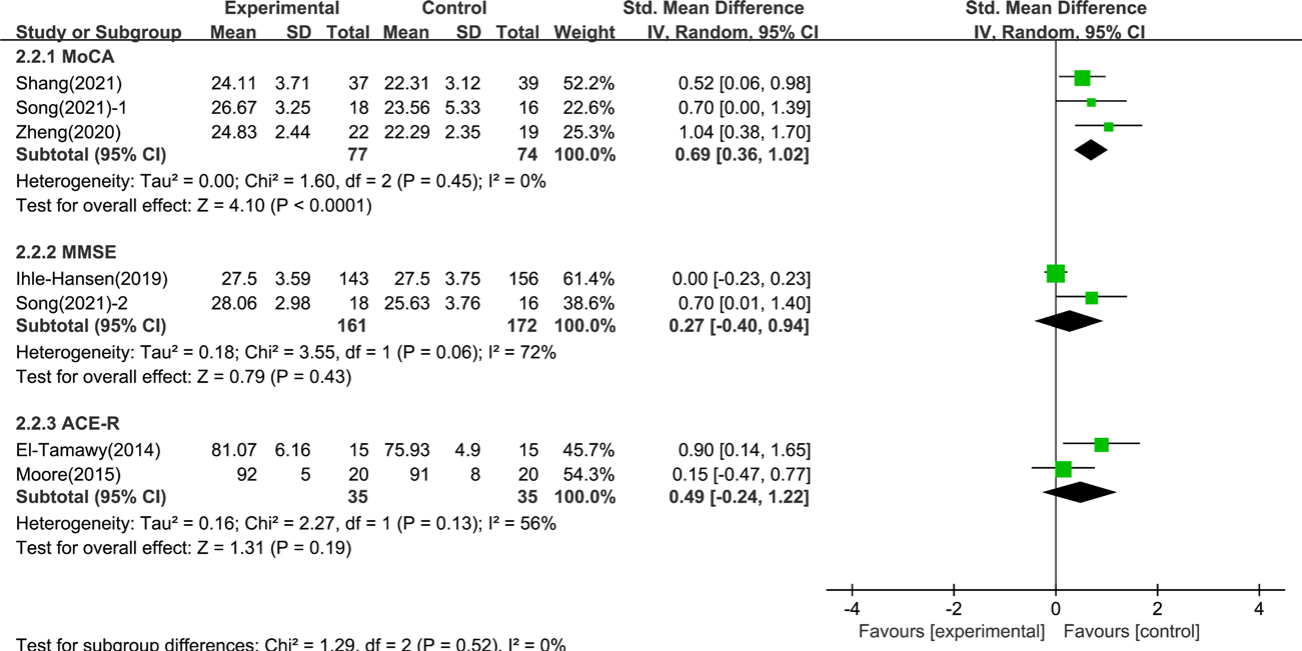
Subgroup analysis of global cognitive function effect size under different measurement tool.

The results of the overall effect heterogeneity test showed that there was heterogeneity among the groups, indicating that the effect of aerobic exercise on cognitive function was affected by potential regulatory variables. Since the included studies involve different exercise intervention intensities and multiple cognitive function measurement tools, it is necessary to conduct subgroup analysis on these 2 variables. According to exercise intensity subgroup analysis (Fig. 4), moderate intensity aerobic exercise intervention had the largest effect size on enhancing global cognition ability (0.98; 95% CI 0.48–1.47; *P* = .0001), followed by low intensity aerobic exercise intervention (0.49; 95% CI 0.10–0.87; *P* = .01), there was no difference in high intensity between the intervention group and the control group (0; 95% CI -0.23 to 0.23; *P* = 1.00). And there was a significant differences among the subgroups (*χ*^2^ = 14.37, df = 2 [*P* = .0008], *I*² = 86.1%). This were shown in Figure 5, we found no significant differences between subgroups through the measurement tool subgroup analysis (*χ*^2^ = 1.29, df = 2 [*P* = .52]; *I*² = 0%).

#### 3.3.2. Specific domains of cognition.

**Figure 6.**
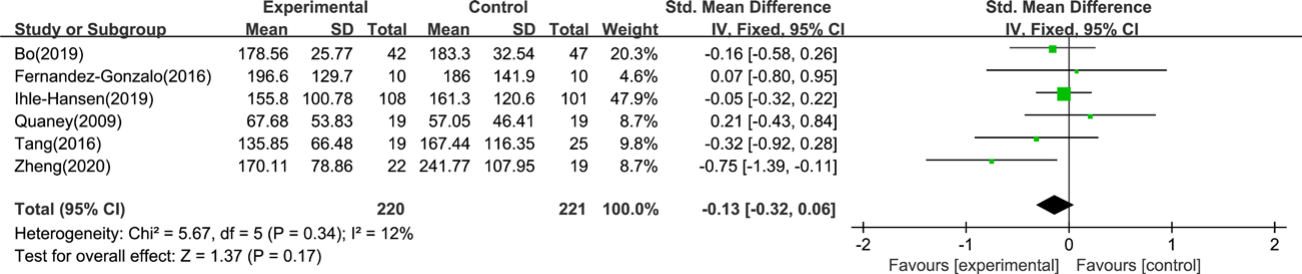
Forest plot showing individual study and pooled effect of aerobic exercise on cognitive flexibility.

**Figure 7.**
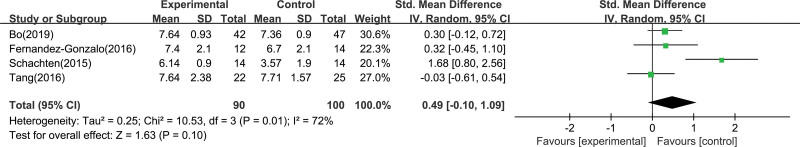
Forest plot showing individual study and pooled effect of aerobic exercise on working memory.

**Figure 8.**
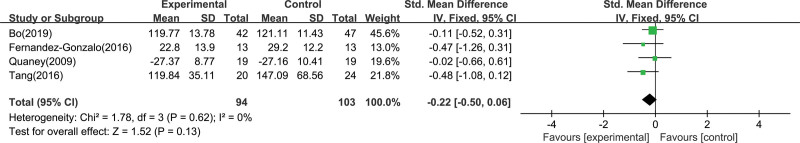
Forest plot showing individual study and pooled effect of aerobic exercise on selective attention and conflict resolution.

We also evaluated changes in special cognitive domains, including cognitive flexibility, working memory, selective attention and conflict resolution. Six studies with 448 participants reported on cognitive flexibility. There was no heterogeneity between 6 research, fixed effect models were used for analysis (Fig. 6), and there was no significant difference between the 2 groups (-0.13; 95% CI -0.32 to 0.06; *P* = .17). Four studies reported the working memory of 190 participants. There was moderate heterogeneity among the 4 studies, and analyzed using a random effects model (Fig. 7), with no significant difference between the 2 groups (0.49; 95% CI -0.10 to 1.09; *P* = .10). Four studies involving 294 participants showed selective attention and conflict resolution. There was no heterogeneity among the 4 research (*I*^2^ = 0%, *P* = .62), and fixed effect models were used for analysis (Fig. 8), and there was no significant difference between the 2 groups (-0.22; 95% CI -0.50 to 0.06; *P* = .13).

## 4. Discussion

Numerous studies have shown that aerobic exercise can control and strengthen cognitive function in patients after a stroke. In this research, meta-analysis was used to analyze the impact of aerobic exercise on the improvement of patients’ cognitive function after stroke from the perspective of evidence-based medicine. The results of the meta-analysis showed that the combined effect of aerobic exercise on global cognition ability was d = 0.51, which significantly improved global cognition ability. Compared with similar studies, the effect size obtained in this study was significantly higher than that in Shu et al’s study (d = 0.13).^[28]^ Shu et al focused on the effects of aerobic exercise on cognitive ability in survivors of ischemic cerebrovascular disease, but not all subjects were stroke patients. In contrast, the 11 articles included in this research only involved stroke patients.

This research also investigated the reasons for the differences in the results of numerous studies through the moderating effect test, including the moderating variables including exercise intervention intensity and measurement method. In terms of the intensity of exercise intervention, this research found that aerobic exercise intervention of various intensity had significant differences in improving patients’ cognitive function after stroke. Moderate-intensity aerobic exercise had the best effect (d = 0.98), followed by low-intensity aerobic exercise (d = 0.49). The effect size of high-intensity aerobic exercise was not statistically significant, which was consistent with Constans et al’s research^[29]^ conclusion. And that’s presumably because cognitive function improvements were concomitant with an increase in hippocampal brain derived neurotrophic factor level, but the high-intensity exercise provoked higher levels of stress-hormone, which might down brain derived neurotrophic factor level in hippocampus.^[30]^ In terms of the measurement method, a moderate effect was observed in the studies using MoCA (d = 0.69), whereas the pooled effect sizes of the studies using MMSE and ACE-R were not significantly different. This result indicates that MOCA may be a better choice of measurement tools when assessing cognitive gains. The reason may be that MoCA has stronger detection sensitivity than MMSE and ACE-R. Evaluation analysis has also been reported in larger sample sizes in the UK,^[31]^ the USA,^[32]^ and Canada.^[33]^ It shows that the sensitivity of MCI in MoCA is about 80%~90%, and the sensitivity of detecting dementia detection is greater than 90%, and compared with MMSE evaluated at the same time, it is significantly improved. Moreover, Breton et al^[34]^ in its meta-analysis showed that MoCA has higher sensitivity and trait for MCI than ACE-R.

In addition, we examined the effects of an aerobic exercise intervention in different cognitive domains such as cognitive flexibility, working memory, selective attention and conflict resolution for improvement. However, none of these aspects showed significant differences. Cognitive flexibility, working memory, selective attention and conflict resolution are important sub components of executive function.^[35]^ Different from the results of this study, some experiments have shown that aerobic exercise can increase the activation level of the left dorsolateral prefrontal cortex, thereby improving executive function.^[36]^ The completion of executive function may depend on the dynamic interaction between prefrontal cortex and other cortex and subcortical regions.^[37]^ However, the brain area on which each sub component of executive function depends has its own focus. For example, the prefrontal lobe plays a key role in the development of cognitive flexibility,^[38]^ and the frontoparietal connection composed of executive control network and dorsal attention network is an important neural basis in the development of working memory ability.^[39]^ Considering the specific role of different regional cerebral cortex in the sub-components of executive function, we suggest that the site of stroke may be an important regulatory variable. In addition, a previous meta-analysis found no significant difference in executive function performance between aerobic exercise and daily physical activity. It believed that gender might be an important factor affecting the effectiveness of aerobic exercise in improving executive function, and women can benefit a lot from aerobic exercise.^[28]^ Similarly, in our study, all the trials included in the analysis were male dominated. Therefore, more large-scale studies are needed to determine the impact of aerobic exercise on executive function, and to determine whether gender and disease site affect the intervention effect.

This Meta-analysis also has some limitations: Initially, only published English literature was included in this survey, which may affect the completeness of the data to some extent. Second, limited by the information in the incorporated literature, this study was unable to determine whether individual characteristics of stroke patients would affect the intervention effect of cognitive function through regulating variables. In the future, more evidence-based intervention studies are needed to analyze the interaction mechanism between individual characteristics of stroke patients, aerobic exercise and cognitive function, and verify the impact of aerobic exercise intervention on the different domains of cognition.

## 5. Conclusion

In summary, it can be said that aerobic exercise has a good effect on improving the cognitive dysfunction of patients after stroke, moderate-intensity exercise is most likely to maximize cognitive gains in stroke patients. Nevertheless, our survey did not find that aerobic exercise had a favorable effect on cognitive flexibility, working memory, selective attention and conflict resolution.

## Author contributions

**Conceptualization:** Xiaogang Li, Di Geng.

**Data curation:** Siyue Wang, Guotao Sun.

**Formal analysis:** Xiaogang Li, Di Geng.

**Investigation:** Guotao Sun, Siyue Wang, Xiaogang Li.

**Methodology:** Di Geng, Xiaogang Li, Guotao Sun.

**Software:** Xiaogang Li, Di Geng.

**Writing – original draft:** Xiaogang Li, Siyue Wang.

**Writing – review & editing:** Di Geng, Xiaogang Li.
